# Impact of the COVID-19 Pandemic on Malaria Control in Africa: A Preliminary Analysis

**DOI:** 10.3390/tropicalmed8010067

**Published:** 2023-01-16

**Authors:** Liping Gao, Qi Shi, Zhiguo Liu, Zhenjun Li, Xiaoping Dong

**Affiliations:** 1 National Institute for Viral Disease Control and Prevention, Chinese Center for Disease Control and Prevention, 155 Changbai Road, Changping, Beijing 102206, China; 2 National Institute for Communicable Disease Control and Prevention, Chinese Center for Disease Control and Prevention, 155 Changbai Road, Changping, Beijing 102206, China; 3 Vocational and Technical College, and College of Veterinary Medicine, Inner Mongolia Agricultural University, Baotou 014109, China

**Keywords:** malaria, epidemiology, COVID-19, disrupt, Africa

## Abstract

Malaria remains a significant public health concern in Africa, and the emerging coronavirus disease 2019 (COVID-19) pandemic may have negatively impacted malaria control. Here, we conducted a descriptive epidemiological analysis of malaria globally, and preliminarily explored the impact of COVID-19 on the malaria elimination program in regions of Africa (AFR). The present analysis found that there was a vast heterogeneity of incidence of deaths caused by malaria globally in different continents, and the highest malaria burden was observed in AFR. In 2020, there was an obviously increasing trend in the malaria epidemic in AFR, while the other four continents exhibited stable and declining patterns. Historically, malaria has been largely concentrated in high-malaria-burden regions, such as West Africa, and there has been an obvious increasing trend in Nigeria. These data suggest that dynamic changes in the malaria epidemic situation worldwide have primarily originated from AFR, and West Africa has played an important role in the global malaria increase in recent years. Under the coercion of COVID-19, multiple factors have co-driven the increase in malaria in AFR, including insufficient financial investments, a high native malaria burden, weak surveillance systems, limited medical resources, and low socioeconomic development levels. In addition, the shift of medical resources (e.g., health workers and personal protective equipment (PPE), the manufacturing of diagnostic reagents, and drugs) from malaria control to emergency COVID-19 response in the pandemic’s early stage caused disruptions, reductions, and delays in pillar malaria control measures, leading to a significant negative impact on malaria control. In particular, a funding shortfall at both the international and domestic levels led to a “significant threat,” resulting in vast gaps in access to proven malaria control tools. Although there has been a declining trend in malaria control over time due to COVID-19, the effect still cannot be ignored. Hence, we recommend the implementation of medical and technical resource assistance as a priority strategy to support Africa (West Africa) in order to curb further transmission.

## 1. Introduction

Malaria remains one of the most virulent and deadliest parasitic diseases in the world, and it is widely distributed in the tropics and subtropics, particularly in Africa and Southeast Asia. It continues to have a devastating impact on the health and livelihood of people, despite being preventable and curable [1,2]. Malaria is caused by the genus *Plasmodium*, which belongs to the apicomplexan phylum and invades erythrocytes [3].

There are four major species of the human malarial parasite: *Plasmodium falciparum* (Pf), *P. vivax* (Pv), *P. malariae* (Pm), and *P. ovale* sp. (Po) [4], of which *Plasmodium falciparum* (Pf) and *P. vivax* (Pv) are responsible for most of the disease burden caused by malaria [5]. Previously, reports have shown that approximately 99% of the malaria-associated mortality worldwide is caused by Pf [6], which kills an estimated 409,000 people annually (1 December 2020) [2] and is a major cause of deaths in the African region [7,8]. Mosquitoes play a significant role in the transmission of malaria, and medically important mosquito species, including *Anopheles gambiae*, are important malarial vectors in Africa [9]. Malaria is mainly transmitted to humans through the bites of *Anopheles* mosquitoes [10]. The main clinical manifestation of malaria is fever, while other common symptoms include chills, nausea, myalgias, headache, and vomiting. Malaria is suspected if a person presents with these symptoms and has recently traveled to an endemic region, or has received intravenous medication or a transfusion [11]. Hence, early diagnosis and treatment are crucial steps to curb deaths.

In the last decade, based on the implementation of effective intervention strategies, including insecticide-treated nets (ITNs), indoor residence spraying (IRS), rapid diagnostics, and drug intervention in children < 5 years of age and pregnant women, malaria control has achieved considerable progress [12].

Quarantine measures, movement restrictions, lockdowns, curfews, and travel bans are some of the most effective response measures that helped the world contain the spread of COVID-19 [13]. It is thought that the measures have led to a resurgence in malaria, which has the potential to become a severe public health concern worldwide [14]. A study showed that there was a 5% global increase in the malaria incidence rate and 12% increase in malaria mortality in 2020 relative to 2019, with 47,000 additional deaths owing to the service disruptions during the COVID-19 pandemic [8], especially in WHO African regions (AFR) [15]. In 2019, Africa accounted for 94% (213 million cases) of malaria cases and 94% (386,000 deaths) of malaria deaths globally [2], the majority of which appeared in the sub-Saharan African region [16]. An epidemiological analysis of the global malaria status will contribute to understanding the change dynamics of malaria in different continents and help us to implement control strategies and allocate medical resources accordingly. In addition, it is important to understand how the COVID-19 pandemic has affected malaria control intervention strategies in high malarial burden regions, such as West African countries, and this will provide helpful information to implement remedial measures to mitigate the high incidence and death rate of malaria. Therefore, the purpose of the current study is to ① perform a descriptive epidemiological analysis of the global malaria situation to preliminarily explore the extent and change dynamic of malarial infections in different continents that contribute to the determination of high malaria regions, ② and to conduct an analysis of the literature of the influence of COVID-19 on malaria control in high malaria burden regions, such as West African countries, to provide insights to better formulate targeted control measures to decrease the effect of COVID-19 on malaria control and stop the further spread of malaria, as well as to optimize the allocation of limited medical resources in these countries.

## 2. Methods and Materials

### 2.1. Study Design and Data Source

The design of our study included a descriptive analysis of the global malaria evolution profile based on global malaria cases and deaths (Figure S1). The low social-economic development level and weak health surveillance capacity caused us to highlight the African malaria burden. We then further explored the COVID-19 effect on malaria control in Africa using multiple accessible analysis factors that provided comprehensive clues for the formulation of targeted control measures for preventing further malaria rebound in West Africa, as well as to optimize the allocation of medical resources in undeveloped countries. In our study, data on confirmed cases of malaria (incidence rate) and deaths (deaths rate) caused by malaria from multiple continents were obtained from the World Health Organization (WHO, the World Malaria Report (2021)) [8]. These data were cross-checked by two trained and qualified health workers and then processed and analyzed using Microsoft Excel (Microsoft Office 2016, Microsoft Corporation, Redmond, WA, USA). Subsequently, based on these data, a preliminary description analysis of the global malaria epidemiology profile was conducted. This included the evolution of the global malaria epidemiology, the geographic distribution profile, the epidemic trends of malaria in each continent, and the dynamic variation of malaria in Africa. All of the valid data were processed and analyzed using GraphPad Prism 5.0 (GraphPad Software Inc., San Diego, California) to illustrate the mapped epidemiology of malaria in each continent.

### 2.2. Literature Research Strategies to Evaluate the Impact of the COVID-19 Pandemic on Malaria Control in Africa

**Figure 1:**
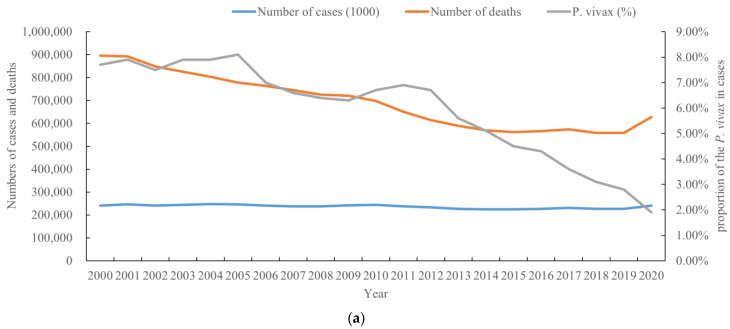

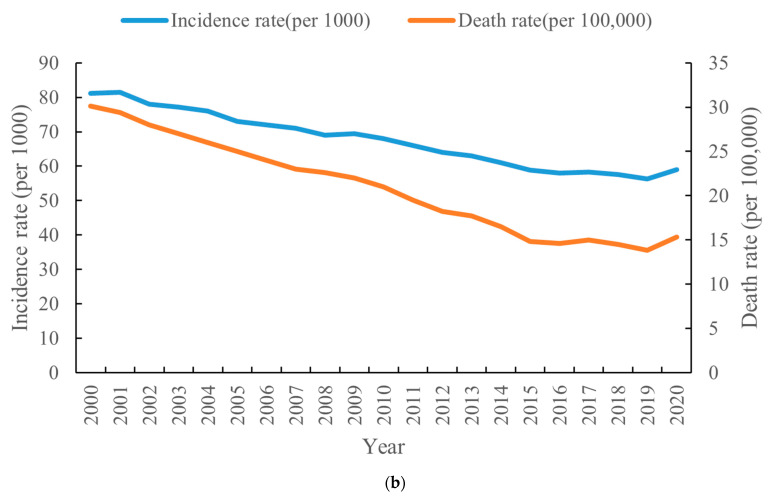
Change trend of the global malaria epidemic from 2000 to 2020. (**a**) Number of malaria cases and deaths; (**b**) incidence rate (1000) and death rate (100,000).

Briefly, the study protocol complied with previous criteria [17], with restrictions to those published in the English language up until 1 June 2022 (Figure 1). Three main search terms were used: “COVID-19,” “Malaria,” and “Africa” for the related document research. Then, the document research was conducted based on the related scholar community platforms, primarily focusing on PubMed, Ovid MEDLINE (R), MedRxiv, and *The Lancet*. A total of 677 related documents were obtained, all of which were downloaded for further analysis. Two authors independently reviewed the titles and abstracts before conducting full text reviews. The documents selected for full-text analysis were assessed for eligibility before inclusion. The authors, title, study population, study place, study design, and findings were extracted from the eligible papers to explore the impact of COVID-19 on malaria control. Following screening and review, 608 documents were removed after the targeted screening, and 33 documents that were not relevant to this topic were excluded. Finally, 36 related documents were selected for final analysis, and they were sorted by study type as follows: modeling (predictive) study, report (e.g., general report, case report), review, opinion paper, and policy guideline. Furthermore, the COVID-19 pandemic disruption to health services and malarial control measures in Africa were analyzed. This analysis included many aspects, such as the financial investment gap, delays and disruption to pillar countermeasures, clinical service disruptions, and medicine resource shortages.

## 3. Results

### 3.1. Epidemic Profile of Malaria at the Global Level, 2000–2020

From 2000 to 2020, the total number of reported malaria cases and deaths globally were 4,965,000,000, and 14,563,000, respectively. The average number of cases of and deaths from malaria annually was at least 248,250,000 and 728,150, respectively. The number of reported cases of malaria fluctuated from 2000 to 2019, followed by an obvious increase in 2020 (Figure 1a). A total of 241 million cases of malaria were recorded in 2020, and there were an additional 14 million cases compared to 2019, leading a serious public health threat. In contrast, the number of deaths due to malaria significantly decreased from 2000 to 2015, and from 2016 to 2019, there was a slight increase in the number of deaths due to malaria. In 2020, the number of deaths increased significantly, which posed challenges in maintaining healthcare services and controlling malaria in this region (Figure 1a). However, the proportion of cases caused by *P. vivax* declined annually, from 7.7% in 2000 to 1.9% in 2020 (Figure 1a). Globally, the incidence and death rates of malaria showed the same clear gradual decreasing trend annually from 2000 to 2015, while there was a slowing and stalling trend during 2015–2019 (Figure 1b). Indeed, the incidence rate (per 1000) declined from 81.1 in 2000 to 56.3 in 2019, and the death rate declined from 30.1 in 2000 to 13.8 in 2019. However, there was a substantial increase in the incidence and death rates in 2020 (Figure 1b), and in particular, there was a visible increase in death rates in 2020. From 2000 to 2015, the number of deaths decreased steadily by 37%, and slowly by 0.7% to 2019, but rose to 627,000, a 12% increase, from 2019 to 2020 (Figure 1a, b).

### 3.2. Distribution of the Number of Cases of and Deaths from Malaria in Different Continents, 2000–2020

**Figure 2:**
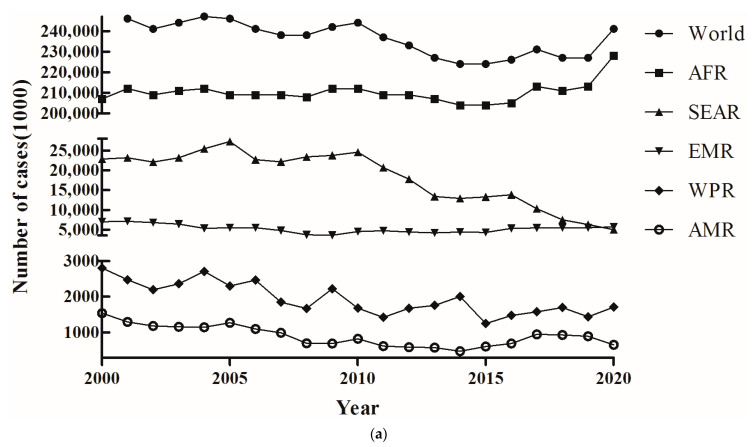

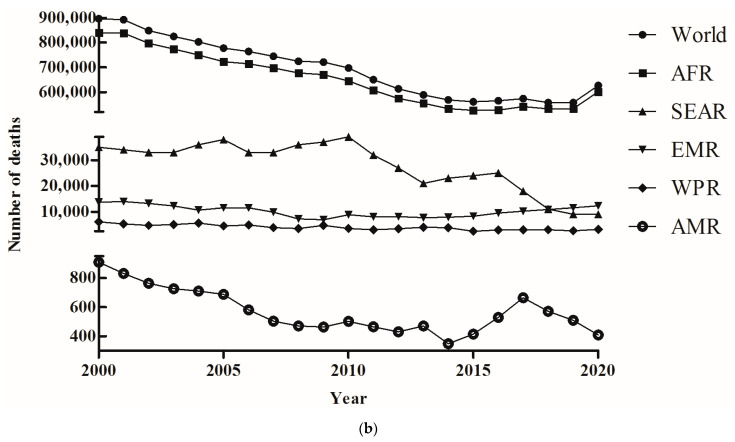
Geographic distribution of the number of cases (**a**) and deaths (**b**) of malaria globally during 2000–2020.

Based on the number of cases, the global malaria epidemic situation was sorted into three levels: low, medium, and high epidemic levels (Figure 2a). The first level contained the lowest number of cases, including 40,728,000 in the WHO Western Pacific Region (WPR), and 17,850,000 in the WHO Region of America (AMR), while the second level was observed in the WHO South-East Asia Region (SEAR) (*n* = 381,900,000) and the WHO Eastern Mediterranean Region (EMR) (*n* = 109,600,000) (Figure 2a). The WHO African Region (AFR) belonged to the third level and harbored the most malaria cases (*n* = 4,413,000,000) from 2000 to 2020 (Figure 2a). The malaria cases of three continents, namely, WPR, AMR, and SEAR, showed an ongoing decreasing trend during 2000–2020, while EMR showed a stable epidemic situation. Although AFR represented the highest malaria burden, it had a relatively stable epidemic situation during 2000–2015. However, there was an obvious increase after 2015, and this led to an upward global malaria case trend. In particular, malaria cases apparently increased in 2020 (Figure 2a). Similarly, the number of deaths were also divided into three levels, where the lowest and highest rates were found in the AMR (*n* = 11,956) and AFR (*n* = 13,669,000), respectively (Figure 2b). The number of deaths in the WPR (*n* = 829,00), SEAR (*n* = 586,000), and EMR (*n* = 213,700) were ranked in the second level (Figure 2b). However, the malaria cases increased by 7%, and deaths due to malaria increased by 12.7% in AFR, which accounted for approximately 95% of cases and 96% of deaths worldwide, respectively. Furthermore, there was a consistent malaria case and death distribution pattern between AFR and the global situation, while four continents exhibited stable and declining trends. These data suggested that dynamic changes in global malaria deaths are primarily related to the situation in AFR (Figure 2a,b).

### 3.3. Malaria Incidence and Death Rate Distribution in Different Continents, 2000–2020

**Figure 3:**
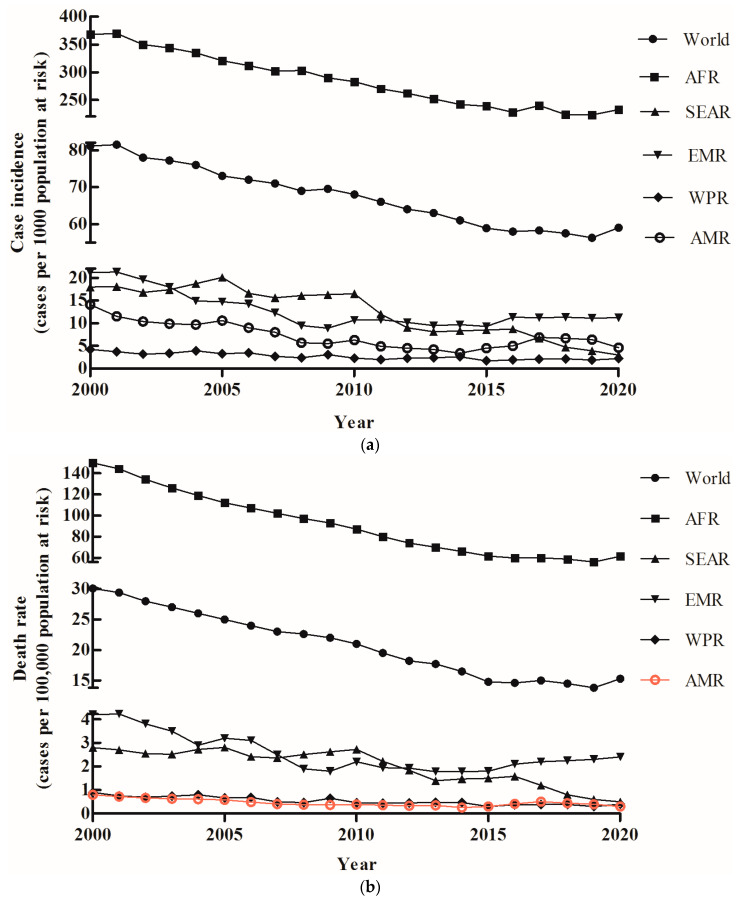
Geographic distribution of the incidence rate (**a**) and death rate (**b**) of malaria globally during 2000–2020.

There was a large difference in the malaria incidence and death rate in five continents. Based on the malaria incidence and death rate, two different epidemic levels were observed in five continents. The highest malaria incidence and death rate were found in AFR, and all the other continents exhibited low malaria incidence and death rates (Figure 3a,b). The lowest malaria incidence and death rates were observed in WPR and MAR, respectively. Although AFR had a high malaria burden, the malaria incidence and death rate declined continuously from 2000 to 2019 and then increased suddenly in 2020 (Figure 3a, b). There was not only the lowest malaria incidence and death rate level in the other four countries, but also the malaria incidence and death rate continued to decrease from 2000 to 2020 (Figure 3a,b). In contrast, the malaria incidence and death rate in EMR, which was the lower malaria burden continent, showed a slight upward trend from 2016 to 2020 (Figure 3a,b). These data demonstrated that the malaria incidence and death rate showed an increasing trend worldwide, and the malaria burden continued to be high in AFR. The global malaria incidence and death rates increased from 14 and 56.3 in 2019 to 15 and 59.0, respectively, in 2020 (Figure 3a,b).

### 3.4. Epidemic Characteristics of Malaria in African Countries, 2000–2020

**Figure 4:**
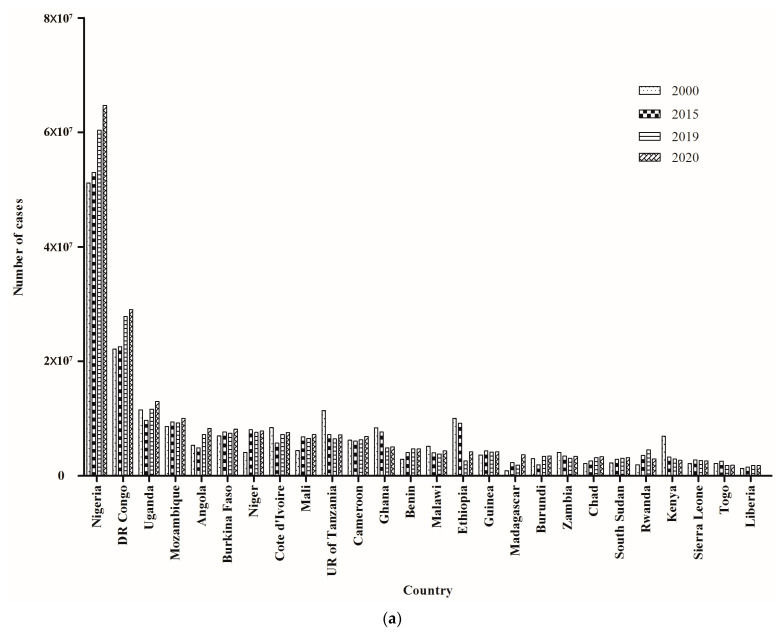

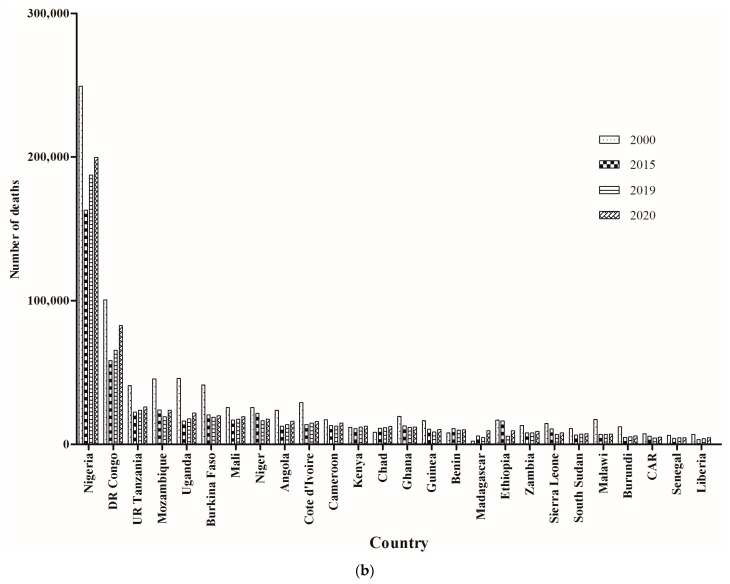
Comparison of the number of cases (**a**) and deaths (**b**) resulting from malaria in African countries across 4 years.

**Figure 5:**
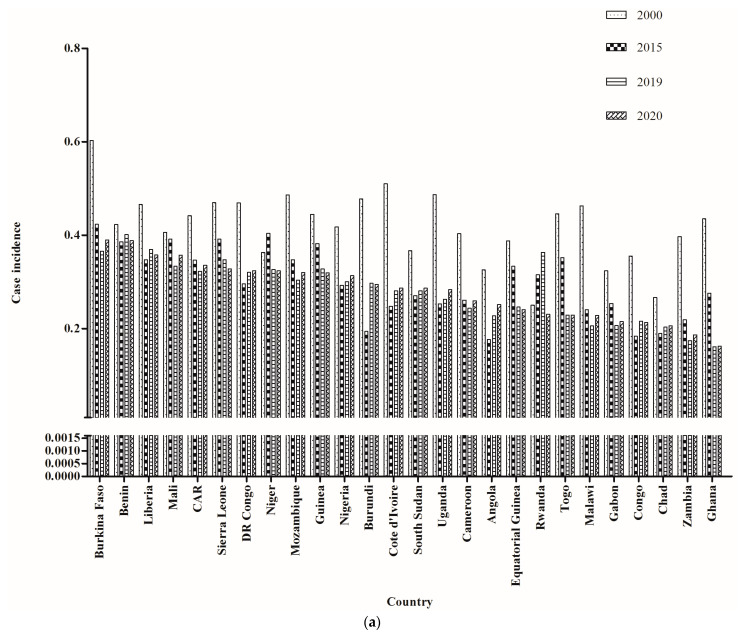

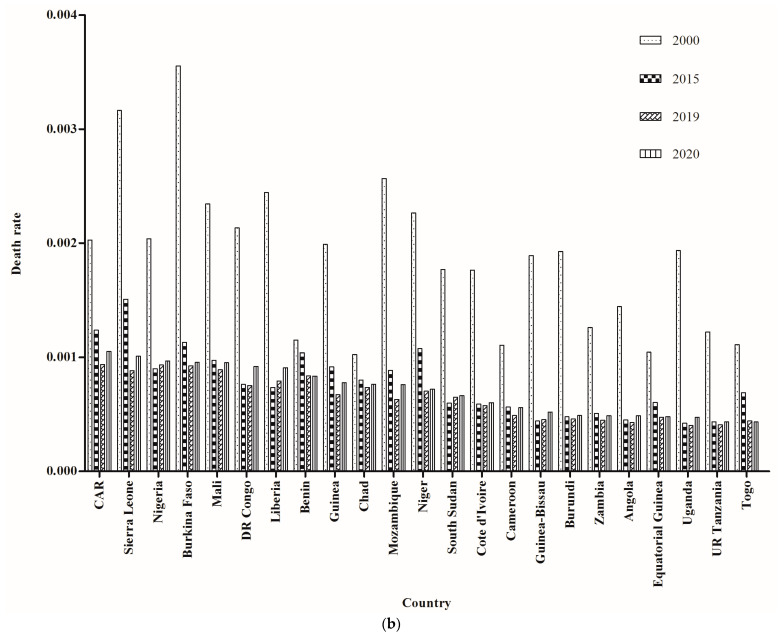
Comparison of the incidence rate (**a**) and death rate (**b**) resulting from malaria in African countries across 4 years.

Although the highest burden of malaria was in AFR, both epidemic situations decreased annually in most countries in AFR from 2000 to 2015 (Figure 4a,b). However, there was an obvious increasing trend in some West African countries after 2015, such as Nigeria and the DR Congo. Moreover, from 2000 to 2020, the majority of malaria cases and deaths in AFR were distributed in West Africa (Figure 4a,b), followed by Central Africa. Additionally, 11 countries from West Africa were among the first 26 countries with the highest malaria case incidence and death rate in AFR from 2000 to 2020. Both the malaria incidence and death rate fell significantly from 2000 to 2015 in most countries, but they had stalling trends from 2015 to 2019. It is worth noting that both the malaria case incidence and death rates appeared to increase from 2019 to 2020, especially in some countries in West Africa (Figure 5a,b). These data suggested that West Africa played an important role in the global malaria increase in recent years.

### 3.5. COVID-19 Pandemic Disruption to Health Services and Malaria Control in Africa

The restriction of movement, intermittent health services, reduced visits to health facilities due to fear of getting infected with and being diagnosed with COVID-19, and the stigmatization of COVID-19 treatment centers have culminated in the ineffective treatment of malaria. Our preliminary analysis showed that the COVID-19 pandemic impacted the health service and malaria control in Africa in multiple ways and in various aspects. The main impacts are the following: investment budget decreases, medical resources shortages, the disruption or delay of pillar control measures, reduced access to healthcare services, reduced and delayed diagnostic and clinical visits, and restrictions on and disruption to the availability of curative and preventive malaria commodities as well as the mental burden. Moreover, vaccine hesitancy (VH) and stigmatization due to COVID-19 vaccines could further affect the introduction and use of the malaria vaccine in Africa.

### 3.6. Gap in Financial Investment in Malaria Control

There was a significant gap in financing malaria control globally, and this imposed a significant burden on the economies of AFR countries. Despite the total investment funding for malaria control and elimination being successively increased from 2018 to 2020, constituting USD 2.7 billion in 2018 [18], USD 3.0 billion in 2019 [2], and USD 3.3 billion in 2020 [8], there is expected to be a financial gap of approximately USD 6.8 billion in 2020 compared to the goal [8]. Furthermore, the proportion of the protected population was reduced annually from 2010 (161 million) to 2015 (127 million), with only 87 million estimated in 2020. Undoubtedly, this vast financial gap primarily occurred in AFR countries, triggering malaria surveillance and control in these regions that showed a slow decline or stall situation from 2015 to 2019. In addition, due to the prevalence of COVID-19, there was an obvious increasing trend in the malaria epidemic in 2020. Malaria control therefore has robust epidemiological and socioeconomic benefits; however, sustained and abundant financing is required to accelerate these gains.

### 3.7. Delays and Disruption to Pillar Countermeasures

Accordingly, the impact of COVID-19 on malaria control was primarily focused on severe impacts to malaria control programs (e.g., ITNs, IRS, the Intermittent Preventive Treatment of Malaria for Pregnant Women (IPTp)) in some lower income countries (Table S1). Previous reports have shown that only 19 countries from West Africa have completed ITN distribution, 11 countries had moderate delays, and the other 9 had substantial delays. Additionally, only 47% of ITNs were distributed in 2021, with 53% still waiting to be delivered to target communities in the future. Moreover, 25 of 37 countries had completed spraying or were on target to complete by 2020. Twelve countries (Angola, Benin, Botswana, Burkina Faso, Burundi, Cabo Verde, Honduras, Mozambique, Somalia, and Zimbabwe) reported a risk of delays in IRS activities, and spray activities in both Namibia and Sudan were seriously delayed. Obviously, delays and disruption to pillar countermeasures occurred in the highest malaria-burdened West African countries, and this was bound to reverse the existing gains.

### 3.8. Clinical Service Disruption during the Pandemic

During September 2020 to June 2021, clinical service disruptions ranged from 4% to 93%, with approximately 30% of community health service points reporting a moderate to substantial level of discontinuity. The reductions in outpatient attendances and malaria testing in the majority of countries were the main effects observed in the early stage of the COVID-19 pandemic and were consistent with the epidemic waves of COVID-19 in West Africa. Additionally, approximately 20% of the 65 malaria-endemic countries experienced partial and (or) severe disruption in malaria diagnosis and treatment. Comparisons of three periods (April to June, July to September, and November to December) in 2019 and 2020 showed that, overall, in nine high-malaria-burden countries, there were 27.13%, 9.44%, and 3.07% reductions in malaria testing, respectively (Table S2). However, comparing January to March between 2020 and 2021 showed a 2.89% increase in malaria testing (Table S1). Currently, due to the intermittent health service disruptions with the multiple waves of COVID-19 transmission, only 5% of the population has been fully vaccinated against malaria in low- and middle-income countries. Clinical service disruption during the pandemic often resulted in rapid malaria resurgence, primarily threatening vulnerable populations (children and pregnant women).

### 3.9. Reduction in Medicine Resources

The early stage of the COVID-19 pandemic was characterized by a reprioritization of malaria-related medical resources for COVID-19 response, including personal protective materials (e.g., personal protective equipment (PPE), gloves, and gowns), experimental materials, reagents, facilities, and health workers, as well as the shortage of logistics and support materials, including fuel supply and generator maintenance. In fact, individuals sometimes hesitate or do not attend health facilities due to a fear of being infected with COVID-19, or because they cannot afford transport fees. In addition, health workers require additional equipment resources to protect themselves from COVID-19, and supplies of diagnostics and drugs are being interrupted. These disruptions greatly aggravate the vulnerable African population (children and pregnant) dying of malaria and directly impact the efforts to control malaria. Moreover, the COVID-19 vaccine shortage and purchase barriers were the primary challenges for the rollout of vaccinations in Africa.

## 4. Discussion

In the present study, we conducted an epidemiological change analysis of the global malaria status and preliminarily explored the impact of COVID-19 on the malaria elimination program in African regions. The present study highlighted that the emergence of the COVID-19 pandemic posed a serious challenge to malaria control in Africa, and multiple socio-economic factors co-drove the increase in the number of cases of malaria in 2020, in which the COVID-19 pandemic may have played a significant role. The immeasurable impact of infectious disease pandemics on health systems could be disastrous, especially when there are similarities in clinical presentations with other diseases [19]. As such, the COVID-19 pandemic has posed an overwhelming burden on the fragile healthcare systems in Africa and has disrupted various health-care services, including malaria prevention and treatment programs [20]. The COVID-19 pandemic has adversely affected the provision and uptake of malaria prevention and treatment services, especially the mass test, treat and track (MTTT) of malaria being implemented at the community level in Ghana [21]. The civil conflict and lack of healthcare services present compounded with the COVID-19 pandemic resulted in a five- to tenfold increase of malarial incidence in the past years in Afghanistan [22]. The prevalence of malaria in Franceville was very high during the COVID-19 pandemic, and this prevalence was the highest compared to the percentage of cases in 2019 (29.6%) and 2021 (31.5%) (*p* < 0.001) [23]. The emergence of the COVID-19 pandemic indirectly caused an increase in the prevalence of malaria and thwarted progress in malaria control [24]. Indeed, the COVID-19 pandemic resulted in disruptions to routine health services and the diversion of already limited available resources in sub-Saharan Africa [25]. Similarly, the Ebola outbreak in Africa was curbed following the distribution of ITNs and antimalarial drugs, but it led to increased malaria spread and dramatic increases in the deaths of children [26]. Remarkably, malaria diagnosis and treatment were affected by COVID-19, with 46% of the 68 countries reporting experiencing disruptions [27], and the average disruptions were reported across outbreak detection and control activities for non-COVID-19 diseases and services for malaria in 36% of the countries [28]. In addition, approximately 30–40% of malaria endemic countries reported different levels of disruption to malaria pillar measures [28]. In India, the pandemics often cause crosscutting disruptions in health care and in the supply of drugs and diagnostics that may have obstructed and delayed treatment and caused loss of lives in cases of complicated malaria and the incidence of cerebral malaria [29]. Therefore, there was an obvious impact of COVID-19 on malaria control that not only caused direct disruption (or delay) of healthcare services, but also could cause considerable indirect economic losses. Although there was early evidence of service recovery, nearly all 129 countries were still affected by the COVID-19 pandemic [30]. However, the revision of standards of campaign implementation during COVID-19, as well as effective coordination supported by real-time decision making through digital data management, have been factors in the success of this campaign [31].

With the exception of the COVID-19 pandemic, the reasons for the stall in malaria control progress are complex and are not limited to the following: insufficient funding, a decline in the coverage of vector control interventions, a majority of people living in high-risk areas, inadequate health care facilities, climatic conditions, and environmental factors in sub-Saharan Africa [32]. First, insufficient finances are a vital risk to malaria control in the highest malaria-burdened countries of sub-Saharan Africa. Historical evidence has demonstrated that nearly all resurgence events can be attributed, at least in part, to the weakening of malaria control programs for a variety of reasons, of which resource constraints are the most common [33]. Several reports have shown that countries with higher investments in health security were better prepared to respond to the COVID-19 pandemic [34]. The gains experienced in the past decade can be attributed, in part, to the increased financing available to scale up effective interventions. The reduced interventions were predicted to cause an additional 38.2 clinical cases, 2500 deaths, and additional economic losses of USD 14.1 billion in Ghana [35]. Health financing trends suggest that substantive policy interventions are required to ensure that malaria elimination is adequately financed and that the available financing is effectively targeted to interventions that provide the best value for money [36]. Malaria vectors have a strong ecological association with rice agroecosystems, which can provide abundant aquatic habitats for larval development [37]. There are a large number of irrigated rice-growing communities that contribute to an increase in the number of mosquitoes in sub-Saharan Africa, resulting in greater malaria infection risk in humans [38]. Furthermore, both small and large dams stand out as high areas of elevated malaria burdens in some regions of SSA [39]. Additionally, insecticide resistance in malaria vectors cannot be ignored in the context of malaria control. Although IRS is vital for controlling malaria by targeting vectors, the continuing potential of IRS serves to increase resistance [40]. Remarkably, the malaria incidence is closely related to seasonal rainfall, and generally, malaria has an incidence peak with a lag of approximately 2 months after the rainy season [41]. A previous study suggested that local evolution of the resistance of malaria vectors to current drugs is an important bio-threat [42], showing an independently emerging artemisinin resistance in Africa, as well as kelch13 mutations, a resistance-conferring gene in *P. falciparum* isolates in Northern Uganda, increasing annually from 3.9% to 19.8% from 2015 to 2019 [43]. In addition, we found that the number of cases caused by *P. vivax* was reduced significantly annually [44]. The *P. vivax* prevalence was lower than *P. falciparum* prevalence in Africa, where the population has a low expression of the Duffy antigen [45], and this may contribute to the increasing infection rates. Africa, particularly West Africa, plays a vital role in the prevalence and spread of malaria globally. Moreover, global warming [46] and environmental factors, including climate, rain, ecology, and human activities, also contribute to the increasing case incidence and death rate due to malaria in West Africa. Recently, there was a growing VH powered by issues related to the management of the response to the COVID-19 pandemic, which may have hampered the acceptance of malaria vaccines across the continent [47]. Unfortunately, the negative impact of COVID-19 has not been restricted to malaria, but has also increased undernutrition, vaccine-preventable pneumonia and diarrhea, tuberculosis, HIV, and sickle cell disease [48]. Owing to the surge in COVID-19, MPXV, and other infections, malaria-control efforts could deteriorate and further worsen [49]. Therefore, these negative impacts can be mitigated by implementing targeted measures. First, we need to widen financial channels to increase budgets, secure the continuous provision of ITNs, implement IRS, and initiate trials for manufacturing mRNA vaccines against malaria [50]. In addition, more cost-effective tools, more rapid and accurate diagnostic methods, information technology, robust logistics, and highly efficient surveillance are required to meet the requirements for malaria control projects [51,52,53]. Second, the preventive treatment for malaria among school-age children [54] and an early targeted intervention for COVID-19 can significantly reduce the impacts on malaria transmission [55].

Although our analysis provides insight into the epidemiology of malaria during the COVID-19 pandemic, several limitations remain. First, our analysis was based on previously published data and documents, and our data were inadequate for performing a systematic review. Hence, the conclusions of this study may be biased. Second, a basic analysis of the relationship between malaria and COVID-19 cases across countries was not performed because accessing the relevant data was a great challenge. Third, due to the inaccessibility of malaria-related data in every country, a systematic review of the evolution of malaria epidemiology over time in different countries is required.

## 5. Conclusions

In the present study, we conducted a preliminary descriptive epidemiology analysis of global malaria and performed an investigation of the effect of COVID-19 on malaria control in Africa. There was a vast heterogeneity in the malaria global incidence and deaths in different continents, and this remains a serious public health issue in undeveloped African countries. Furthermore, the numbers of malaria cases and deaths showed increasing trends in 2020, and the most serious situation was observed in AFR. In particular, the West African countries had the highest malaria burden. Our analysis suggests that the COVID-19 pandemic has a negative impact on malaria control and prevention programs in various aspects [24]. These include reduced and delayed diagnostic and clinical visits, reduced access to healthcare services, medical resource shortages, disruption or delay of pillar control measures, and disruption to the availability of curative and preventive malaria commodities. Although the effect of COVID-19 on malaria control is difficult to accurately quantify and calculate, further investigation is necessary to comprehensively assess the impact of the COVID-19 pandemic on malaria control in West Africa and to tailor countermeasures. Our analysis highlights that improved diagnostic capacity, strengthening of the medical resource supply chain, enhancing investment in the R&D of new vector control tools and insecticides, and more effective medicines must be prioritized.

## Data Availability

The data presented in this study are available on request from the corresponding author.

## Supplementary Materials

The following supporting information can be downloaded at: https://www.mdpi.com/article/10.3390/tropicalmed8010067/s1, Figure S1. Flowchart of the study design. Table S1. Comparisons change trends in four different periods in malaria test performed. Table S2. The impact of COVID-19 pandemic on malaria control in Africa.
